# Spatial resolved transcriptomics reveals distinct cross-talk between cancer cells and tumor-associated macrophages in intrahepatic cholangiocarcinoma

**DOI:** 10.1186/s40364-024-00648-z

**Published:** 2024-09-11

**Authors:** Zhao-Ru Dong, Meng-Ya Zhang, Ling-Xin Qu, Jie Zou, Yong-Heng Yang, Yun-Long Ma, Chun-Cheng Yang, Xue-Lei Cao, Li-Yuan Wang, Xiao-Lu Zhang, Tao Li

**Affiliations:** 1 Department of General Surgery, Qilu Hospital, Shandong University, Jinan, 250010 Shandong China; 2 Department of Physiology and Pathophysiology, School of Basic Medical Sciences, Cheeloo College of Medicine, Shandong University, Jinan, 250012 Shandong China; 3 Department of Geriatrics, Qilu Hospital, Shandong University, Jinan, 250012 Shandong China; 4 Department of Clinical Laboratory, Qilu Hospital, Cheeloo College of Medicine, Shandong University, Jinan, 250010 Shandong China; 5 Department of Hepatology, Cheeloo Hospital, Cheeloo College of Medicine, Shandong University, Jinan, 250012 Shandong China

**Keywords:** Intrahepatic cholangiocarcinoma, Tumor associated macrophage, Trefoil factor 3 (TFF3), Spatial transcriptomics, Digital spatial profiler

## Abstract

**Background:**

Multiple studies have shown that tumor-associated macrophages (TAMs) promote cancer initiation and progression. However, the reprogramming of macrophages in the tumor microenvironment (TME) and the cross-talk between TAMs and malignant subclones in intrahepatic cholangiocarcinoma (iCCA) has not been fully characterized, especially in a spatially resolved manner. Deciphering the spatial architecture of variable tissue cellular components in iCCA could contribute to the positional context of gene expression containing information pathological changes and cellular variability.

**Methods:**

Here, we applied spatial transcriptomics (ST) and digital spatial profiler (DSP) technologies with tumor sections from patients with iCCA.

**Results:**

The results reveal that spatial inter- and intra-tumor heterogeneities feature iCCA malignancy, and tumor subclones are mainly driven by physical proximity. Tumor cells with TME components shaped the intra-sectional heterogenetic spatial architecture. Macrophages are the most infiltrated TME component in iCCA. The protein trefoil factor 3 (TFF3) secreted by the malignant subclone can induce macrophages to reprogram to a tumor-promoting state, which in turn contributes to an immune-suppressive environment and boosts tumor progression.

**Conclusions:**

In conclusion, our description of the iCCA ecosystem in a spatially resolved manner provides novel insights into the spatial features and the immune suppressive landscapes of TME for iCCA.

**Supplementary Information:**

The online version contains supplementary material available at 10.1186/s40364-024-00648-z.

## Background

As a highly lethal hepatobiliary malignancy, intrahepatic cholangiocarcinoma (iCCA) is increasing in incidence and has low survival rates [1]. Accumulating evidence suggests that iCCA features a dense stromal reaction and complex tumor immune microenvironment, composed of immune cells, cancer-associated fibroblasts (CAFs), and tumor-associated macrophages (TAMs) involved in tumor progression [2, 3]. However, the complicated interaction landscapes of iCCA cells and multiple stromal components are not entirely understood, especially in a spatially resolved way. TAMs dominate the immune cell population in the tumor microenvironment (TME) of most solid tumors [4, 5]. The phenotype and metabolism of macrophages are affected by intense communication with other cells in the TME via cell-cell contact-dependent mechanisms and soluble messengers. By stimulation, macrophage polarization ranges from pro-inflammatory and anti-inflammatory/immune-suppressive activation. Targeting TAMs is a favored immunotherapy strategy. Further investigations are needed to elucidate the underlying molecular crosstalk mechanisms driving iCCA pathogenesis to identify novel therapeutic targets.

Histopathology is exclusively used as a diagnostic tool in clinical settings, as many diseases are characterized by abnormal spatial organization within tissues [6]. Tissue transcriptomes are usually studied using either bulk RNA-sequencing (RNA-seq) or single-cell RNA sequencing (scRNA-seq) approaches, neither of which reserve tissue spatial information [6]. Molecular heterogeneity, within and between tumors, has been recognized in most malignant tumurs [7–10]. Expansion of tumor subclones with varying molecular alterations within and beyond the entity greatly influences disease evolution and therapeutic effects [11–13]. ScRNA-seq is a powerful tool for transcriptionally dissecting cellular variability within the tissue. It has been applied in previous studies to delineate the inter-tumor heterogeneity of human iCCAs and identify four malignant epithelial subclusters with distinct marker genes [10]. However, the positional context of heterogenous gene expression containing information regarding pathological changes and cellular variability in iCCA is lacking. For this purpose, the spatial transcriptomics (ST) method and digital spatial profiler (DSP) technology were developed to decipher the spatial architecture of variable tissue cellular components in multiple physiological or pathological scenarios [14–17].

Here, we aimed to define the transcriptome of specific areas and decipher iCCAs’ spatial inter- and intra-tumor molecular heterogeneity by applying spatial-resolved high-throughput technologies for in situ and quantitative gene expression detection. By retaining the tissue domains’ positional information, we could superimpose the malignant variability and expression data onto tissue histological images, making the transcriptomics spatially visible. In addition, we depicted the interaction landscape between malignant cells and TAMs that are close to one another. The TFF proteins secreted by iCCA cells could reprogram macrophages into a tumor-permissive state with high S100A protein expression levels.

## Methods

### ICCA samples and RNA quality control

Three human iCCA samples were obtained from the Department of general surgery of Qilu Hospital (Jinan, China). This study was conducted in accordance with the Declaration of Helsinki with written informed consent from all patients. The project was approved by the Regional Ethics Committee at Shandong University, Jinan, China. The tumor samples were embedded in OCT (#4583, SAKURA) before cryo-sectioning for RNA extraction. A 10 μm section for each patient was placed in Lysing Matrix D (#116913050, MP Biomedicals) and lysed using FastPrep (MP Biomedicals). Total RNA was extracted and RIN values were determined. Sections underwent successfully steps only if passed the quality control. Hematoxylin and eosin-stained images were manually annotated by a trained pathologist to identify different tissue regions, including tumoral, stromal and normal hepatocellular regions.

### Spatial transcriptomic library preparation and sequencing

Spatial transcriptomic amplification and library preparation were performed by Annoroad Gene Technology (Beijing, China) using Visium Spatial Gene Expression platform (10x Genomics) according to the manufacturer’s instructions. Final libraries were sequenced on Illumina NovaSeq6000 system (Illumina, Inc., San Diego, California, US). Around 3,000 tissue domains in each section were captured and analyzed.

### Pre-processing of raw data

Raw data was processed with *Space Ranger* (10x Genomics). The forward read contained the spatial barcode and UMI sequence while the reverse read contained the transcript information used for mapping to the reference GRCh38 human genome with *STAR* [18]. Afterwards data was processed individually using the R package *STUtility* [19] and normalized using the regularized negative binomial regression method implemented in the *SCTransform* function. The number of variable genes selected with *SCTransform* was determined by applying a residual variance cutoff of 1.1 (variable.features.rv.th = 1.1) with the additional parameter settings: return.only.var.genes = FALSE and variable.features.n = NULL. Individual data was also processed with *Seurat* [20] R package.

### Spot annotation and spatial mapping of single-cell data using SPOTlight

We annotated each spot combining the pathological morphology with canonical marker genes and the R package *SPOTlight* [21] which is centered around a seeded non-negative matrix factorization (NMF) regression, initialized using cell-type marker genes and non-negative least squares (NNLS) to subsequently deconvolute ST capture locations (spots). For this purpose, scRNA-seq dataset (GSE138709) of iCCA samples were downloaded from the Gene Expression Omnibus (GEO) database. We used the same labels as in the figures from original publication [14]. By using annotated single-cell data in combination with ST data, *SPOTlight* estimates proportions of every cell type that presents in the single-cell data at each spatial capture location [21]. Integration was performed by the wrapper function *SPATAwrappers::inferSpotlight()*.

### Marker detection

Differentially expressed genes within each annotated zone were determined using the *FindAllMarkers* function in *Seurat* by performing a Wilcoxon signed-rank test. The function performs the test pairwise between each cluster and its background (all other spots in the data set). Gene markers was filtered to include genes with an adjusted *p* value lower than 0.05 and an average log fold change higher than 1.0, thus omitting down-regulated genes.

### Receptor-ligand interaction inference

Signaling crosstalk via soluble and membrane-bound factors among different spot zones and between macrophages and malignant subclones was predicted using the R package *CellChat v2*[22], which is specially designed for enabling the inference of cell-cell communication from multiple spatially resolved transcriptomics datasets. *CellChat v2* is based on expanded database CellChatDB v2 by including more than 1000 protein and non-protein interactions (e.g. metabolic and synaptic signaling) with rich annotations.

### Digital spatial profiler library preparation and sequencing

The GeoMx Digital Spatial Profiler (DSP) is a method for highly multiplex spatial profiling of proteins or RNAs on fixed sections by counting unique indexing oligonucleotides assigned to each target of interest [23]. In the current study, DSP was performed by CapitalBio Technology Corporation (Beijing, China) as previously described [24]. Following probe hybridization, UV cleavage, and barcode collection, gene expression was quantitated by PCR amplification and Illumina sequencing. The antibodies used in the study were anti-PanCK (#NBP2-33200, Novus) (Cy3, 5.5 µg/ml), anti-CD3 (#sc-20047AF594, SantaCruz) (Texas Red, 10 µg/ml) and anti-CD68 (#sc-20060AF647, SantaCruz) (Cy5, 1 µg/ml).

### Pseudo-time analysis using monocle

We learned trajectory graphs and performed pseudo-time analysis of all malignant subclones from individual patient using R packages *Monocle* [25], demonstrating the evolution trajectories of cancer cells and distinct highly expressed genes along the pseudo-time.

### Cell culture

Cholangiocarcinoma cell lines QBC-939 and HUCCT1, human leukemia monocytic cell line THP-1 were maintained in DMEM media or RPMI-1640 media (Thermo-Fisher Scientific) supplemented with 10% fetal bovine serum (FBS) (Atlanta Biologics), 1% penicillin-streptomycin and 2mM L-glutamine. All cell lines were confirmed mycoplasma-free (MycoAlert PLUS mycoplasma detection kit, Lonza). THP-1 cells were treated with 100ng/ml phorbol-12-myristate-13-acetate (PMA) for 24 h to allow cell adhesion to the plate and differentiation into resting M0 macrophages. For RNA analysis, cells were seeded in 12-well plates at appropriate numbers (5 × 10^4^ – 3 × 10^5^ cells per well) to allow cells to grow to ~ 90% confluence at the endpoint. For siRNA treatment, cells were seeded 48 h before being treated with *TFF3* siRNA or control siRNA. For the coculture experiments, THP-1-derived M0 macrophage cells were placed into the bottom wells of 6-well plate (Corning, NY) at a density of 10,000 cells per cm [2]. To obtain TAMs, M0 were cultured by the addition of conditioned media from CCA cell lines (HUCCT1 or QBC939) for another 48 h.

### Quantitative RT-PCR (qPCR)

Cells were seeded in 12-well plates at the density required to approach confluence at the end of experiment before being lysed for RNA extraction using RNeasy Mini Kit (Qiagen). cDNA was created using qScript XLT cDNA Supermix (Quantabio) per the manufacturer’s protocol. qPCR was carried out using Power SYBR Green Master Mix (Applied Biosystems) and primers (Supplemental Table 1) in an Applied Biosystems StepOne Real-Time PCR cycler: 95 °C (15 s) and 60 °C (1 min). Ct values were calculated using StepOne software accompanying the real-time cycler.

### Whole cell extracts and immunoblotting analysis

Cells grown and treated in 12-well plated were washed with cold PBS twice and then lysed in RIPA lysis buffer (50mM Tris-HCl, pH8.0; 150mM NaCl; 5mM EDTA; 0.5mM EGTA; 1% Igepal CA-630 (NP-40); 0.1% SDS; 0.5% Na deoxycholate) supplemented with 1x protease/phosphatase inhibitor cocktail (#78438, Thermo-Fisher Scientific), 2mM Na_3_VO_4_ and 10mM NaF. Protein concentrations were determined using the BCA protein assay (Beyotime). Lysate samples with the same amount of total protein (40–50 µg) were mixed with 4x Laemmli Sample Buffer (Bio-Rad, with 2-mercaptoethanol) and run on 4–20% Express-Plus PAGE gels in Tris-SDS running buffer (GenScript). Proteins were transferred to PVDF membranes, blocked with 5% non-fat milk and incubated with primary and then secondary antibodies.

### Immnunohistochemistry

Formalin-fixed paraffin-embedded tissue sections from excised specimens were processed for immunohistochemistry (IHC) according to standard procedures. The antibodies used for IHC were anti-CD163 (#16646-1-AP, Proteintech), anti-S100A4 (#16105-1-AP, Proteintech), anti-S100A8 (#15792-1-AP, Proteintech) and anti-TFF3 (#23277-1-AP, Proteintech). IHC staining was performed according to the manufacturer’s instructions. ImageJ software was used to relatively quantify the IHC results. The AOD (Average Optical Density) values of positively stained area was calculated by using a color deconvolution for separating the staining components (diaminobezidine and hematoxylin). All sections were independently analyzed in a double-blind manner by two students, with discrepancies resolved through re-confirmation and results reviewed by a clinician.

### Multiplexed immunofluorescence assay

Tissues were fixed in 4% paraformaldehyde for 24 h, transferred to 30% sucrose for 20 h, embedded in OCT (#4583, SAKURA) and frozen at -80 °C; 10 μm thick slices were rinsed with PBS followed by blocking in 5% serum, 2% BSA, and 0.3% Triton X-100 in PBS. For multiplexed immunofluorescence (mIF) staining, we followed the Opal protocol staining method as previously described [26]. Slides were incubated in primary antibodies at 4 °C overnight, rinsed with PBS, incubated in secondary antibody for 2 h, and mounted with DAPI mounting media. Sections were imaged using a Zeiss M1 fluorescent microscope. Images were processed using Fiji’s ImageJ software. The antibodies used for mIF were anti-PanCK (#NBP2-33200, Novus) (Cy3, 5.5 µg/ml), anti-CD3 (#sc-20047AF594, SantaCruz) (Texas Red, 10 µg/ml) and anti-CD68 (#sc-20060AF647, SantaCruz) (Cy5, 1 µg/ml); or anti-CD163 (#16646-1-AP, Proteintech) (Cy5.5, 1 µg/ml), anti-S100A4 (#16105-1-AP, Proteintech) (SpRed, 2.5 µg/ml), anti-S100A8 (#15792-1-AP, Proteintech) (SpGreen, 2.5 µg/ml) and anti-TFF3 (# 23277-1-AP, Proteintech) (SpOrange, 3.5 µg/ml).

### Statistics

Macrophage differentiation, qPCR, western blot, immunohistochemistry and immunofluorescence assays were done with at least three technical and biological replicates. Data was shown as mean ± SEM. The parametric 2-tailed Student *t* test was used to compare two groups. *P* < 0.05 was considered as statistically significant.

### Data transparency

All the sequencing data related to the clinical samples described in this study have been deposited in the Genome Sequence Archive in National Genomics Data Center under the accession number: HRA11087. The raw sequencing data are available for non-commercial purposes under controlled access because of data privacy laws, and access can be obtained by request to the corresponding authors. All other datasets used and/or analyzed during the current study are available within the manuscript and its supplementary information files. The source code for bioinformatics analyses can be accessed via: https://github.com/xiaolu369/ST-iCCA/blob/main/ST_code.

## Results

### Spatial architecture landscape of iCCA

We included three iCCA cases in the current study. Table 1 summarizes the clinical characteristics of all patients. Around 3,000 spots were captured from each section. We utilized a manual histologic annotation based on a hematoxylin and eosin (HE) image for each case, performed by a trained pathologist, to identify the tumor region and stroma area. The pathological annotation was used for comparison with results from the ST analysis results and for guiding region selection using DSP.

**Table 1:** Clinical characteristics for enrolled patients

Sample ID	Gender	Age	Tumor grade	TNM	Spot counts	Median genes/spot	Median UMI/spot	Total genes
Patient 1	Male	56	G2	T2N0M0, II	3,618	3,209	8,328	21,897
Patient 2	Female	63	G2	T2N1M0, IIIB	3,205	4,998	15,515	22,086
Patient 3	Male	75	G1	T1aN0M0, IA	4,699	5,115	20,399	22,729

**Fig. 1:**
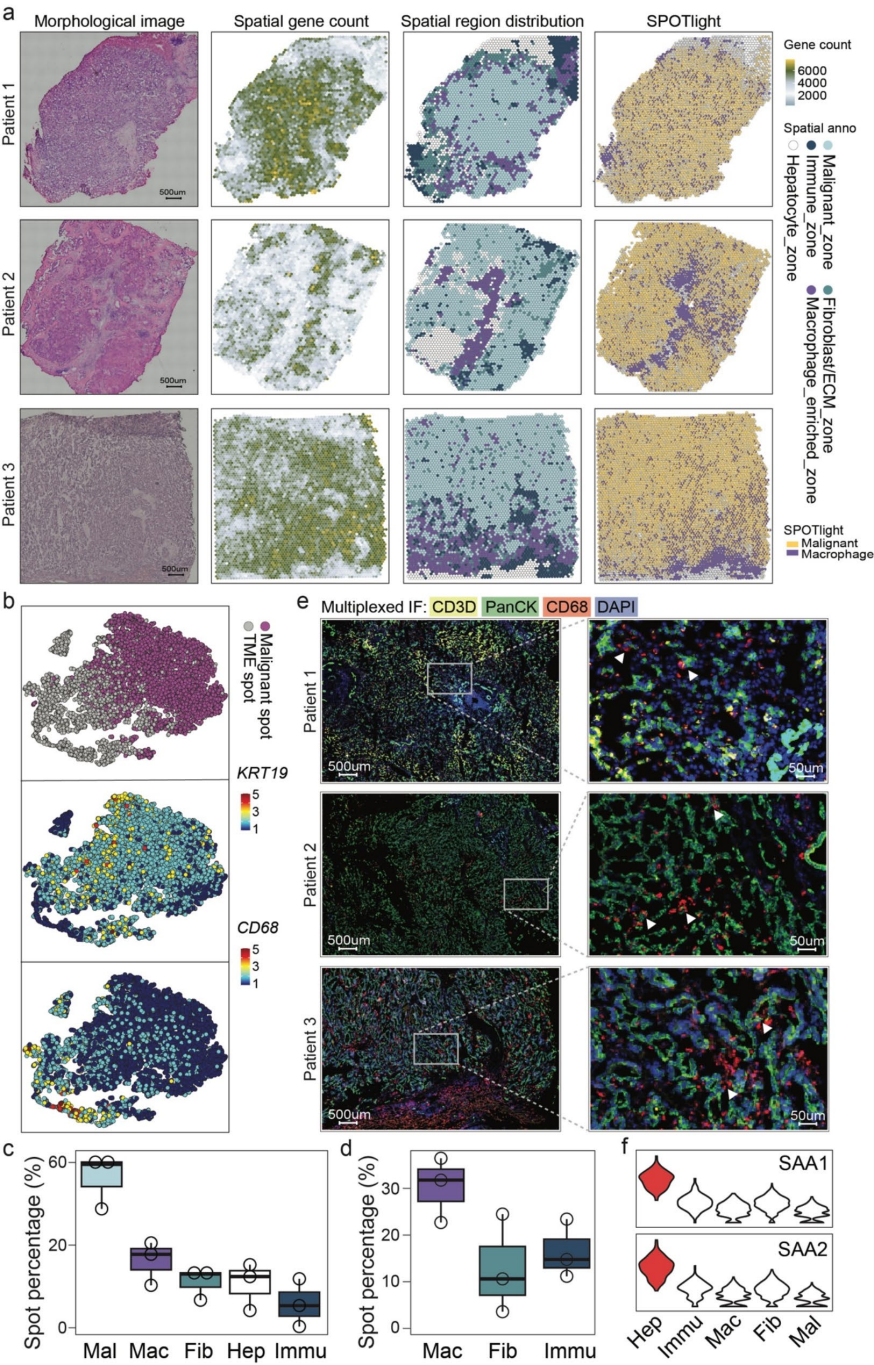
Dissecting iCCA ecosystem by spatial transcriptomics RNA sequencing. (**a**) The spot annotation of ST data and HE staining of tissue sections for three iCCA patients (Patient 1/2/3) enrolled in the current study. (**b**) T-distributed stochastic neighbor (t-SNE) embedding of all spots colored by spot annotation as either malignant spot or TME spot (upper panel), *KRT19* expression level (middle panel) representing for malignant cell and *CD68* expression level (bottom panel) for macrophage. (**c**) Spot proportion of each annotated cell type. Mal: malignant; Mac: macrophage; Fib: fibroblast; Hep: hepatocyte; Immu: immune. (**d**) Spot proportion of malignant spots dominated by specific TME components (macrophage, fibroblast or immune cell). (**e**) Multiplex IF staining of PanCK, CD3 and CD68 on patient tissue sections, the white arrows indicate for CD68 positive macrophages. (**f**) Average expression of *SAA1* and *SAA2* in annotated spot subpopulations

Accordingly, sections of the three iCCA specimens underwent ST library preparation and sequencing. Analyses using *STUtility* tool and annotations of transcriptional signatures identified five spot zones in all slides, including the malignant_zone (56.27%, featured by *KRT19*), macrophage_enriched_zone (14.72%, featured by *CD68*), hepatocyte_zone (10.61%, highly expressed with *ALB*), immune_zone (9.41%, highly expressed with *CD3D*, *MS4A1*, and *MZB1*) and fibroblast/ECM_zone (8.99%, featured by *ACTA2*) (Fig. 1a-c and Supplementary Fig. 1).

All of the distinct zones were mapped to discrete locations. From a spatial view, the majority were malignant_zone spots, which dominated the central part of the sections. The normal hepatocytes wrapped around the section as adjacent liver tissue. The most infiltrated cell type in the TME was the myeloid linage macrophage, while there were few tumor-infiltrating T cells and B cells at the edges. This was consistent with previous findings in which iCCA was not a group of immunogenic malignancies (Fig. 1a-c). However, each patient still had distinct gene expression profiles and spatial architecture, demonstrating the coexistence of multiple cholangiocarcinoma signatures within a single tumor type. The proportion of all spatial zones in individual sections showed a similar distribution (Supplementary Fig. 1b).

As the ST data indicated, macrophages were the most abundantly infiltrated TME component. To further confirm this finding, we applied the *SPOTlight* tool using the single-cell RNA-seq data from GSE138709^14^ (Supplementary Fig. 2a) to deconvolute the ST spots and estimate the proportions of every cell type in the single-cell data at each spatial capture location (Fig. 1a right panel and Supplementary Fig. 2b). As a single ST spot covers around ten cells, so we selected the spots that were annotated as malignant and counted the proportion of dominated TME components. This indicated that the highest proportion was macrophage-dominated malignant spots (32.15%). The fibroblast-dominated and immune cell-dominated spots were 10.08% and 14.04% respectively (Fig. 1d).

Next, we used the multiplexed immunofluorescence (mIF) approach to demonstrate that CD68 positive macrophages were strongly infiltrated in the TME compared to the weak infiltration of CD3D positive immune cells (Fig. 1e). This suggests a macrophage-highly-infiltrated immunophenotype of iCCA. Regarding the surrounding normal hepatocytes, a recent paper [27] identified an invasive zone around the iCCA tumor border containing a subpopulation of damaged hepatocytes with increased serum amyloid A1 (SAA1) and A2 (SAA2) expression. This could lead to macrophage recruitment and M2 polarization. Correspondingly, we confirmed high SAA expression in the hepatocyte zone (Fig. 1f), which could contribute to the high infiltration of macrophages in the TME. We would further explore the mechanisms of macrophage infiltration and their functions in the TME of iCCA.

### Inter- and intra-patient molecular and spatial heterogeneity of iCCA

**Fig. 2:**
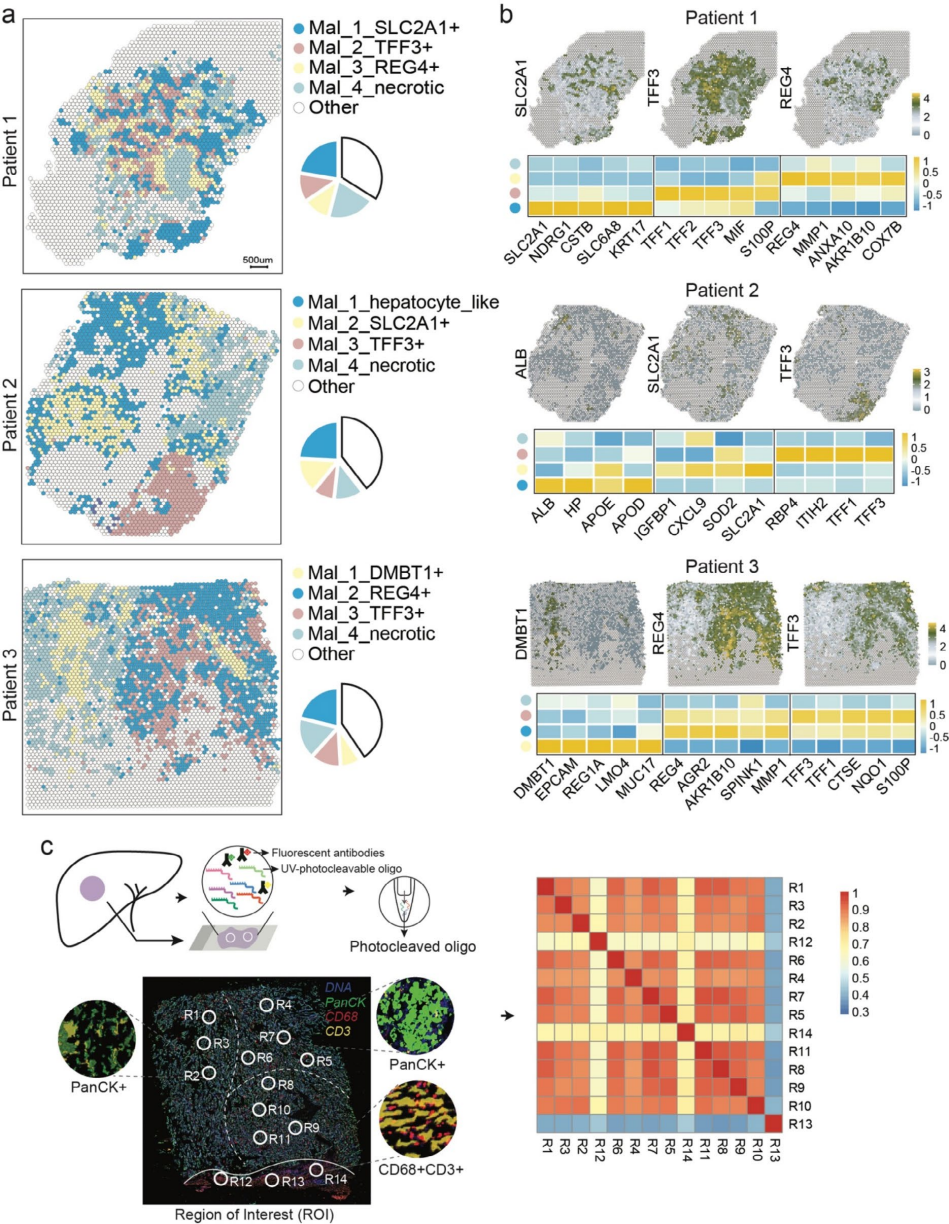
Spatial intra-tumor heterogeneity of iCCA. (**a**) Spatial distribution and proportion of malignant sub-populations in iCCA tissue section. (**b**) Different expressed genes for each malignant sub-population and the spatial expression distribution for typical marker genes. (**c**) DSP sequencing of 14 ROIs on section of patient 3. Left panel showing a schematic overview of DSP workflow and the spatial distribution of ROIs. Antibodies are covalently bonded to a DNA indexing oligo with a UV photo-cleavable linker. The solid line indicating the boundary between malignant enriched region and the stroma enriched region, and the dashed line indicating the boundaries of malignant sub-populations revealed by ST data. Right heatmap showing the overall transcriptional similarities of all ROIs

As inferred by other ST studies on tumors [28], the prominent molecular inter-tumor heterogeneity for dominant cancer cells as somatic mutations are patient-specific [29, 30]. Therefore, we extracted all malignant spots in our data for further analysis to properly capture each patient’s spatial molecular profile. The malignant spots could be further divided into several subclones. Specifically, in patient 1, the malignant spots were divided into four subclones: Mal_1_SLC2A1+, which expressed high levels of *SLC2A1*, Mal_2_TFF3+, with high levels of secreted trefoil factor proteins (*TFFs*), Mal_3_REG4+, which featured high levels of *REG4*, and Mal_4_necrotic, demonstrating a necrotic phenotype. Similarly, we identified four subclones for patient 2 and patient 3, respectively, including a hepatocyte-like subgroup (Mal_1_hepatocyte_like), Mal_2_SLC2A1+, Mal_3_TFF3+, and Mal_4_necrotic for patient 2, and Mal_1_DMBT1+, Mal_2_REG4+, Mal_3_TFF3+, and Mal_4_nacrotic for patient 3 (Fig. 2a, b and Supplementary Fig. 1a). Each malignant subclone expressed distinct cancer-related genes (Fig. 2b and Supplementary Fig. 3). *SLC2A1* is overexpressed in various tumors, which could promote tumor glycolysis, proliferation, and migration [31–34]. A newly published study reveals *SLC2A1* promotes immune suppression and desert of liver metastatic lesions via promoting SPP1 + macrophages and their inhibitory interactions with T cells [35]. *REG4* is also up-regulated in multiple cancer types [36, 37]. The REG4 secreted by cancer cells promotes macrophage polarization to M2, promoting tumor growth and distant metastasis [38]. Necrosis is a pervasive feature of many aggressive, fast-growing tumors, as indicated by the association between iCCAs, phagocyte recruitment, TME modification, and increased risk of tumor metastasis [39].

From a spatial view, the malignant subclones showed distinct distribution patterns, either twisted together, like in patient 1 or driven by physical proximity and separated by stroma regions liker in patient 2 and 3, indicating the malignancy was affected by the surrounding microenvironment, and tumor cells with TME components shaped the intra-sectional heterogenetic spatial architecture. To confirm the spatial distribution features captured by ST data, we further applied the DSP method on sections adjacent to the one used for ST from patient 3. We stained the section with fluorescently labeled antibodies specific to epithelial cell marker PanCK, macrophage marker CD68, and leukocyte marker CD3. We manually isolated regions of interest (ROI) positive for PanCK or CD68 and CD3, which represented regions dominated by malignant cells or macrophages and T cells, respectively (Fig. 2c left panel). The cellularity of a typical ROI averaged ~ 300 cells. We used the negative probe counts to set a quantitation limit [40]. A correlation analysis of all ROIs based on the individual regional transcriptomic profile demonstrated a spatial proximity similarity pattern (Fig. 2c right panel), consistent with the manual demarcation by the dashed lines according to the spatial expression profile of the ST data.

### The spatial expression of TFFs in iCCA

**Fig. 3:**
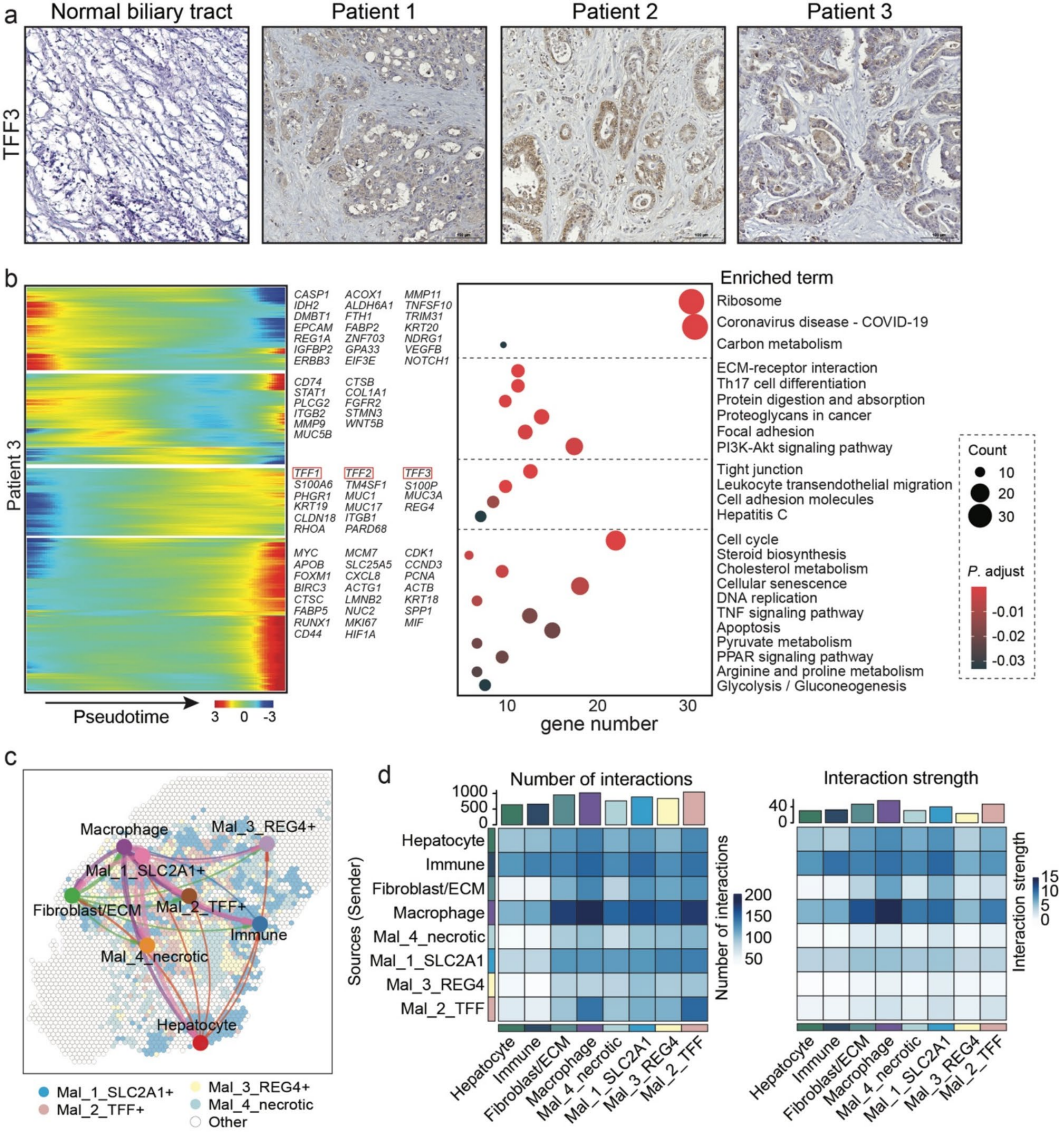
TFF proteins are overexpressed in iCCA and dominantly mediate the crosstalk between malignant cells and macrophage. (**a**) IHC staining showing an intensive expression of TFF3 in iCCA tissues. (**b**) Genes varying along the pseudo-time and the enriched signaling pathways for patient 3. (**c**) Interactions between every two spot groups in patient 1. (**d**) Interaction number and strength between every two spot groups in patient 1

In our data, distinct patients with iCCA shared a common malignant subclone, which highly expressed *TFFs*. The TFF protein family includes three members: TFF1, TFF2, and TFF3, all of which are *mitogens.* TFF3 can stimulate epithelial cell migration in various systems [41]. Previous evidence demonstrated that *TFFs* were involved in the tumorigenesis of cholangiocarcinoma, playing vital roles in the early or in situ tumor stages [42]. As TFF3 is more abundant than the other two proteins (TFF2 is usually undetectable, and TFF1 was significantly more expressed in benign tumors), we mainly focused on TFF3 expression in this study. We first detected TFF protein expression in our iCCA cohort using IHC staining with the TFF3 antibody, which confirmed the prominent distribution of TFF3 in the tumor region compared with the normal biliary tract (Fig. 3a).

Next, to preliminarily explore the function of TFFs in the evolution of cholangiocarcinoma malignancy by ST data, we constructed the pseudo-time trajectory of all malignant spots and visualized the genes varying along the pseudo-time. Unsupervised clustering of those genes and gene ontology (GO) analysis revealed the dynamic transcriptional programs associated with tumorigenesis (Fig. 3b). TFF expression was sustained through the pseudo-time period in all three cases, indicating TFF involvement in malignant tumor evolution. *TFFs* and other genes in the same cluster were enriched in the ‘*tight junction*,’ ‘*cell adhesion*,’ and ‘*HIF-1 signaling*’ pathways (Fig. 3b and Supplementary Fig. 3b).

To get an overview of the cell-to-cell communication landscape in a spatially resolved manner, we used the *CellChat_v2* tool with an expanded database, including more than 1,000 protein and non-protein interactions. The overall profiles presented comprehensive crosstalk between every two annotated spot zones in all three iCCA cases, with differing interaction intensities (Fig. 3c and Supplementary Fig. 4a). Further analysis regarding the receptor-ligand interaction number and strength revealed that the macrophage group exerted the most robust interactions with others, in terms of number and intensity. Interestingly, the TFF + malignant subgroup showed a more active interaction with the macrophages than other malignant subpopulations, either as the sender or recipient (Fig. 3d and Supplementary Fig. 4b, c). This indicates a complicated underlying crosstalk mechanism between TFF-secreting tumor cells and the phenotype of macrophages in the TME. Further investigation is warranted.

### The tumor-permissive activation states of macrophages in the TME of iCCA

Macrophages are extremely versatile and adopt many different activation states or phenotypes in response to their environment [43]. Macrophages residing in the TME are TAMs. TAMs show a tumor-permissive phenotype that is not initiated by lymphocyte signaling but is achieved by the TME [44]. In our spatial data, macrophages were the most aggregated TME cell cluster penetrating the malignancy, contributing to an immunosuppressive microenvironment. We investigated the cross-talk between TAMs and malignant cells to identify any feedback signals.

**Fig. 4:**
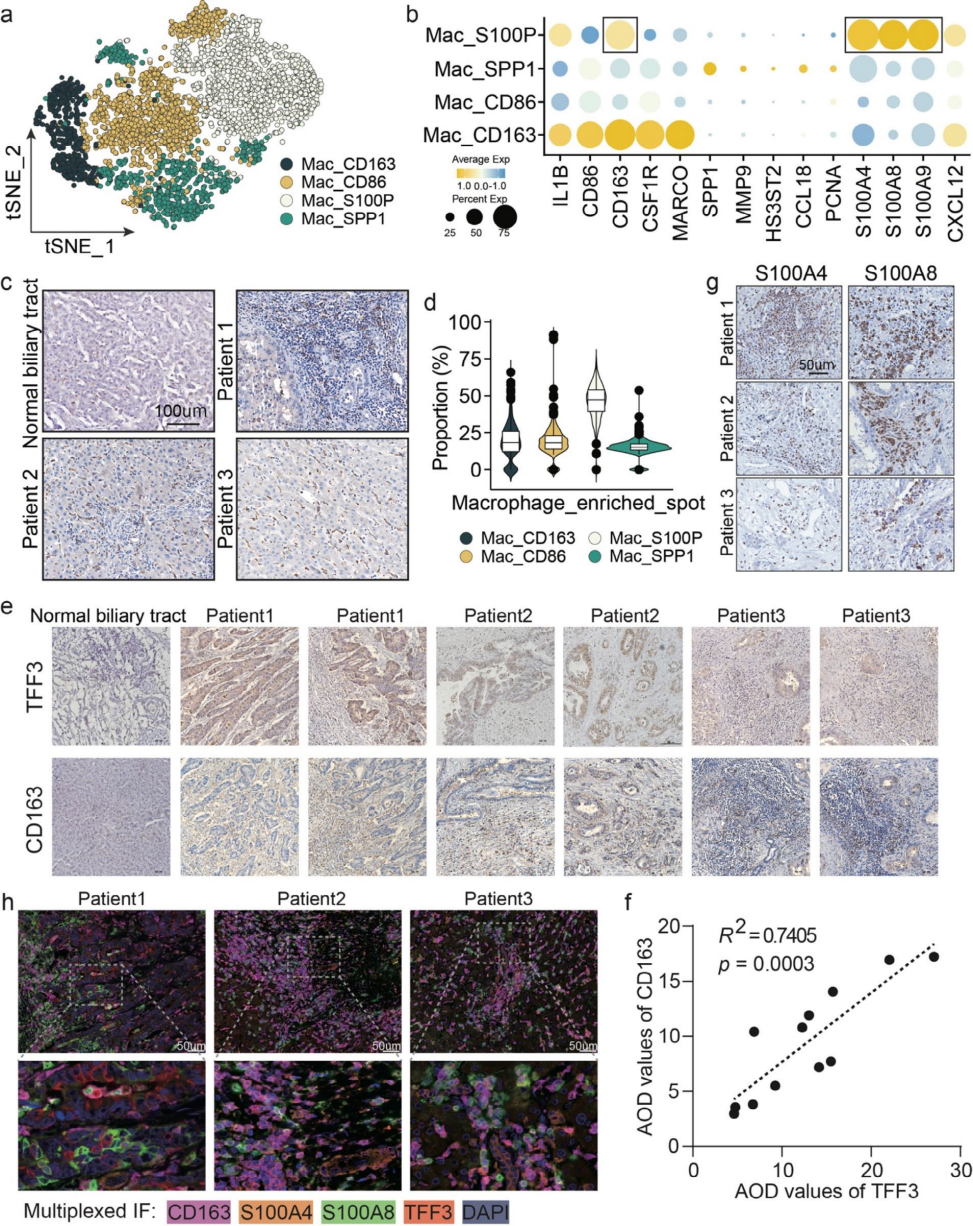
Physical proximity distribution of CD163 + TAMs with TFF3 + malignant cells. (**a**) tSNE plot of macrophages of single cell data, colored by subclusters. (**b**) Dot plots showing the expression levels of marker genes of macrophage subclusters. (**c**) IHC staining showing the expression of CD163 in iCCA tissue indicating for the distribution of CD163 + TAMs. (**d**) *SPOTlight* tool inferring proportion estimate of each macrophage sub-population from the single cell data for the macrophage_enriched spots in ST data. (**e**) IHC staining showing the physical proximity distributions of CD163 and TFF proteins in the same or adjacent microscopic views. (**f**) Linear correlation of AOD values of TFF3 and CD163. (**g**) IHC staining of S100A4 and S100A8 for three iCCA patients. (h) Multiplexed IF staining of CD163, S100A4, S100A8 and TFF3

First, to explore the heterogenous phenotypes of TAMs in iCCA, single cell data of iCCA patients was obtained from GSE138709^14^. We extracted the macrophages for sub-clustering (Fig. 4a). All macrophages were annotated into four sub-populations, each featured by specifically high-expressed marker genes (Fig. 4a, b). Mac_CD163 demonstrated the classical TAM markers *CSF1R* and *MARCO* and the classical M2 marker *CD163*. Mac_SPP1 cells showed positive levels of the novel TAM markers *SPP1* and *MMP9*. Strikingly, the majority of the sub-population was Mac_S100P, which expressed high levels of *S100As* genes (*S100A4*, *S100A8*, and *S100A9*) and *CD163* (Fig. 4b). Recent studies reveal that a monocyte lineage with high expression of *S100A* family genes exerts immunosuppressive function by blocking the CD4 + T cell immune response [45]. All TAM subsets have varying levels of the chemokine *CXCL12*, which is involved in tumor proliferation, immune inhibition by exclusion of NK cells and T cells from tumor areas, [46–49] and homing of cancer cells to metastasis-prone tissues [50].

As *CD163* is a universal marker for macrophages infiltrated in iCCAs and its expression indicates alternative macrophage polarization with a tumor-permissive phenotype, we attempted to confirm its universally spatial distribution in our data (Fig. 4c). We found an immunosuppressive microenvironment in iCCAs. Next, we adopted the *SPOTlight* tool to deconvolve the macrophage_enriched spots in our spatial data to estimate the proportion of each macrophage sub-population from the single cell data. This indicated the dominant macrophage subset was Mac_S100P (Fig. 4d). When we quantified the expression of TFF3 and CD163 by choosing around ten visual fields under a microscope for each patient section (Fig. 4e and Supplementary 5a, b), we further observed a positive linear co-distribution of CD163 and TFF3 (Fig. 4f). Furthermore, we included an additional independent cohort of 24 iCCA patients to perform IHC of TFF3 and CD163, among which, tissue sections of 16 patients showed positive staining of TFF3 with positive of CD163 in physical proximity (Supplementary 5c, d). S100A4 and S100A8 staining confirmed their high expression in the macrophage group (Fig. 4i). We then applied a multiplexed IF staining method, which demonstrated the co-localization of CD163, S100A4, and S100A8 surrounding the TFF3-positive malignant cells (Fig. 4j and Supplementary Fig. 6a). We assumed that the malignant iCCA cells promoted the recruited macrophages towards a tumor-permissive direction by secreting TFF3 proteins. Those alternatively activated macrophage states expressed high levels of S100A proteins terminally towards the M2 polarization.

### Interactions between iCCA malignant cells and macrophages

**Fig. 5:**
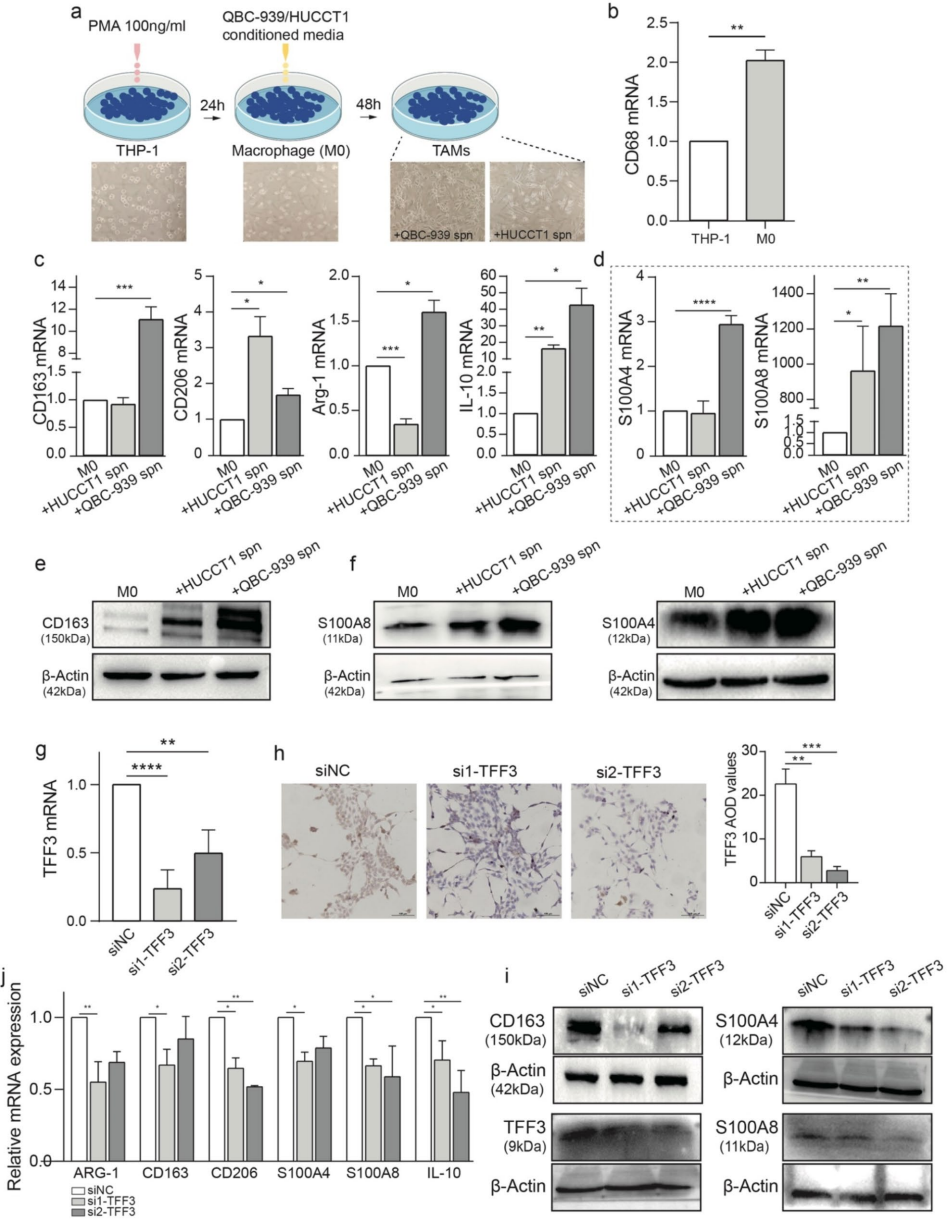
TFF3 secreted by malignant cells promoted the pro-tumor polarization of TAMs. (**a**) Schematic overview of THP-1 stimulation workflow. (**b**) The relative expression level of *CD68* mRNA in THP-1 cell and stimulated M0 cell. (**c**) The relative expression levels of canonical marker genes of M2 after co-culture of cholangiocarcinoma cells with M0 cells. (**d**) The relative expression levels of *S100P* genes after co-culture of cholangiocarcinoma cells with M0 cells. (**e**) The relative protein level of CD163 after co-culture of cholangiocarcinoma cells with M0 cells. (**f**) The relative protein levels of *S100P* genes after co-culture of cholangiocarcinoma cells with M0 cells. (**g**) The relative expression level of *TFF3* after depletion of TFF3 with siRNA in QBC-939 cells. (**h**) IHC staining showing the relative protein level of *TFF3* after depletion of TFF3 with siRNA in QBC-939 cells. (**i**) Western blotting showing the relative protein level of TFF3 with siRNA in QBC-939 cells and CD163 and S100P in TAMs after depletion of TFF3. (j) The relative mRNA expression levels of canonical marker genes of M2 and S100P after depletion of TFF3 with siRNA in QBC-939 cells

To validate our hypotheses, we cultured human monocyte cell line THP-1 and cholangiocarcinoma cell lines QBC-939 and HUCCT1 for an in vitro analysis. First, we used 100 ng/ml of PMA to induce THP-1 monocytes to differentiate into macrophages, as previously reported [51]. After 24 h of incubation, we observed a different cell geometry (Fig. 5a middle panel) and elevated mRNA expression for the macrophage marker CD68 (Fig. 5b). This indicated macrophage differentiation (M0) of THP-1 cells. Then, we co-cultured the supernatant of QBC-939 or HUCCT1 cells with the unstimulated M0 cells. After 48 h, M0 cells exhibited the prominent, elongated shape of M2 polarization [52] (Fig. 5a right panel). To confirm the macrophage polarization state, we performed RT-PCR (Fig. 5c, d) and Western blotting (Fig. 5e, f) to evaluate M2 or TAM marker expression. We found that macrophages stimulated with the supernatant of cholangiocarcinoma cells expressed high levels of M2 markers, including *CD163*, *CD206*, *ARG-1*, and *IL-10* (Fig. 5c, e and Supplementary Fig. 6b). After stimulation, they also up-regulated the expression of *S100A4* and *S100A8* at both the mRNA and protein levels (Fig. 5d, f), consistent with the ST data. This suggests that iCCA malignant cells promote pro-tumor macrophage reprogramming.

Next, to explore whether the above phenotypic polarization is partly mediated by TFF proteins, we designed two siRNAs targeting *TFF3* to attenuate its expression in QBC-939 cells (Fig. 5g-i). After TFF3 depletion, the expression of M2 markers (*CD163*, *CD206*, *ARG-1*, and *IL-10)* and *S100A4* and *S100A8* were remarkably downregulated (Fig. 5i, j), which confirmed our hypotheses that iCCA cells induce the M2 phenotype and pro-tumor polarization of resident macrophages by secreting TFF proteins.

## Discussion

Intrahepatic cholangiocarcinoma is a highly heterogeneous cancer. However, knowledge regarding its tumor microenvironment remains limited, especially in a spatially resolved manner. Here, we provided a spatial atlas of human iCCA at a high resolution to better understand the topological architecture of iCCA heterogeneity. We applied high-throughput ST and DSP technologies to retrieve spatial transcriptomics down to almost an individual cell level, and identified a condensed localization of cancer cells with relatively low levels of TME cell infiltration. Furthermore, we explored the inter- and intra-tumor heterogeneity, considering the tumor subclone organization architecture. The spatial subclones of malignant cells from different patients with iCCA were similar, although inter-tumor heterogeneity was noted. Subclones within the same tumor section were shaped by physical proximity and the surrounding microenvironment. Our data revealed complicated spatial intra-tumor malignant cell compositions of iCCA that were not evident through morphologic annotation.

Traditional therapies such as radiation and standard chemotherapy (gemcitabine and cisplatin) are mostly ineffective for iCCAs and do not significantly improve overall survival [53]. As a complex ecosystem, TME can provide a niche that favors tumor growth, metastasis, chemoresistance, and tumor-specific immune evasion. However, knowledge regarding the tumor stroma interaction in iCCA is lacking. Previous studies have reported that up to 50% of iCCA cases displayed an immune desert pattern characterized by very weak expression of TME signatures. Approximately 20% of cases were characterized by expression of monocyte-derived signatures [54]. Our study provided a comprehensive molecular analysis of the TME for iCCA, which revealed low immune cell infiltration in iCCA ecosystems outside of cancer cores. Moreover, we demonstrated that TAMs play a crucial role in TME-mediated immunosuppressive microenvironment in iCCA ecosystems. TAMs are negative prognostic factors for iCCAs [55, 56], which could explain the poor prognosis and high relapse rate of human iCCA cases.

TAMs stimulate tumor angiogenesis, suppress tumor immunity, and assist with tumor cell migration and invasion [57, 58]. Increased macrophage density in tumor sites is correlated with poor patient survival in many types of cancer. Cancer cell signaling influences macrophage function, while the TAMs-produced proteins promote tumor growth, establishing a feedback loop. TME cells often face oxygen and nutrient deprivation. Hypoxic and nutrient stress provokes tumor cell death, shown as a necrotic zone in our data. This serves as a communicative system attracting macrophages and directing their phenotype [59]. As an aggressive malignancy, hypoxia, nutrient deprivation, and cell death are ubiquitous in iCCAs. This clarifies why we annotated a necrotic zone in all patient cases and detected *HIF-1 signaling* activation sustaining the iCCA tumorigenesis. Interestingly, studies have demonstrated that macrophages in hypoxic areas show more tumor-permissive activation [60].

Macrophages show highly plastic features, and can alter their phenotype in response to environmental signals, which results in fundamentally distinct subpopulations. Pro-inflammatory cytokines, such as interleukin (IL)-1β, IL-6, and IL-8, can classically activate macrophages to the M1 phenotype, mounting an anti-tumor immune response [61]. Hypoxic tumor cells produce cytokines such as oncostatin, HMGB-1, TGF-β, or IL-6 to promote an alternative macrophage (M2) polarization and tumor progression [62]. Other novel genes up-regulated in tumor cells, like *SLC2A1* and *REG4*, promote M2 polarization. In our data, we identified TFF proteins as another group of factors up-regulated by iCCA cells that promote the M2 tumor-permissive polarization of TAMs. Blocking the cross-talk between malignant cells and macrophages by targeting TFF proteins may restrain the suppression of immune surveillance, increase the immune response against tumors, inhibit iCCA progression, and improve patient prognosis. Our data is based on the Visium 10X technology, meaning that the transcriptome data is obtained from a collection of cells within each spot, not individual cells. This method may mask the heterogeneity between cells. However, Visium 10X technology can elucidate the positional relationships and interactions between cells in space.

## Conclusions

In conclusion, our study provides evidence of tumor ecosystem heterogeneity in iCCAs regarding immune suppression and tumor progression, in addition to the crosstalk between iCCA cells and TAMs. Our results indicate that high TFF3 expression in iCCA may promote macrophage polarization towards the TAMs-M2 phenotype. TFF3 may be a potentially important molecule for blocking the crosstalk between TAMs and iCCA cells. In summary, our data help clarify the mechanisms of tumor-macrophage interaction and offer new perspectives for targeting TFF3 in iCCA treatment.

## Electronic Supplementary Material

Below is the link to the electronic supplementary material.

Supplementary Material 1

Supplementary Material 2

## Data Availability

All the sequencing data related to the clinical samples described in this study have been deposited in the Genome Sequence Archive in National Genomics Data Center under the accession number: HRA11087. The raw sequencing data are available for non-commercial purposes under controlled access because of data privacy laws, and access can be obtained by request to the corresponding authors. All other datasets used and/or analyzed during the current study are available within the manuscript and its supplementary information files. The source code for bioinformatics analyses can be accessed via: https://github.com/xiaolu369/ST-iCCA/blob/main/ST_code.

## Abbreviations

CAFs: Cancer-associated fibroblasts
DSP: Digital spatial profiler
GO: Gene ontology
HE: Hematoxylin and eosin
iCCA: Intrahepatic cholangiocarcinoma
mIF: Multiplexed immunofluorescence
RNA-seq: RNA-sequencing
scRNA-seq: Single-cell RNA sequencing
ST: Spatial transcriptomics
TAMs: Tumor-associated macrophages
TFF3: Trefoil factor 3
TME: Tumor microenvironment
