# Supplementary material for: Spatial resolved transcriptomics reveals distinct cross-talk between cancer cells and tumor-associated macrophages in intrahepatic cholangiocarcinoma
Source: Biomark Res. 2024 Sep 11;12:100. doi: 10.1186/s40364-024-00648-z (PMC11389341; doi:10.1186/s40364-024-00648-z)
Supplement: Supplementary file 1 — Supplementary Material 1 [file 40364_2024_648_MOESM1_ESM.docx]

**Supplementary Fig. 1**. (a) Uniform manifold approximation and projection (UMAP) plots of spot clusters for the enrolled iCCA patients. (b) Proportions of spot clusters. (c) Feature plots showing the spatial expression distribution for canonical marker genes of each annotated clusters.


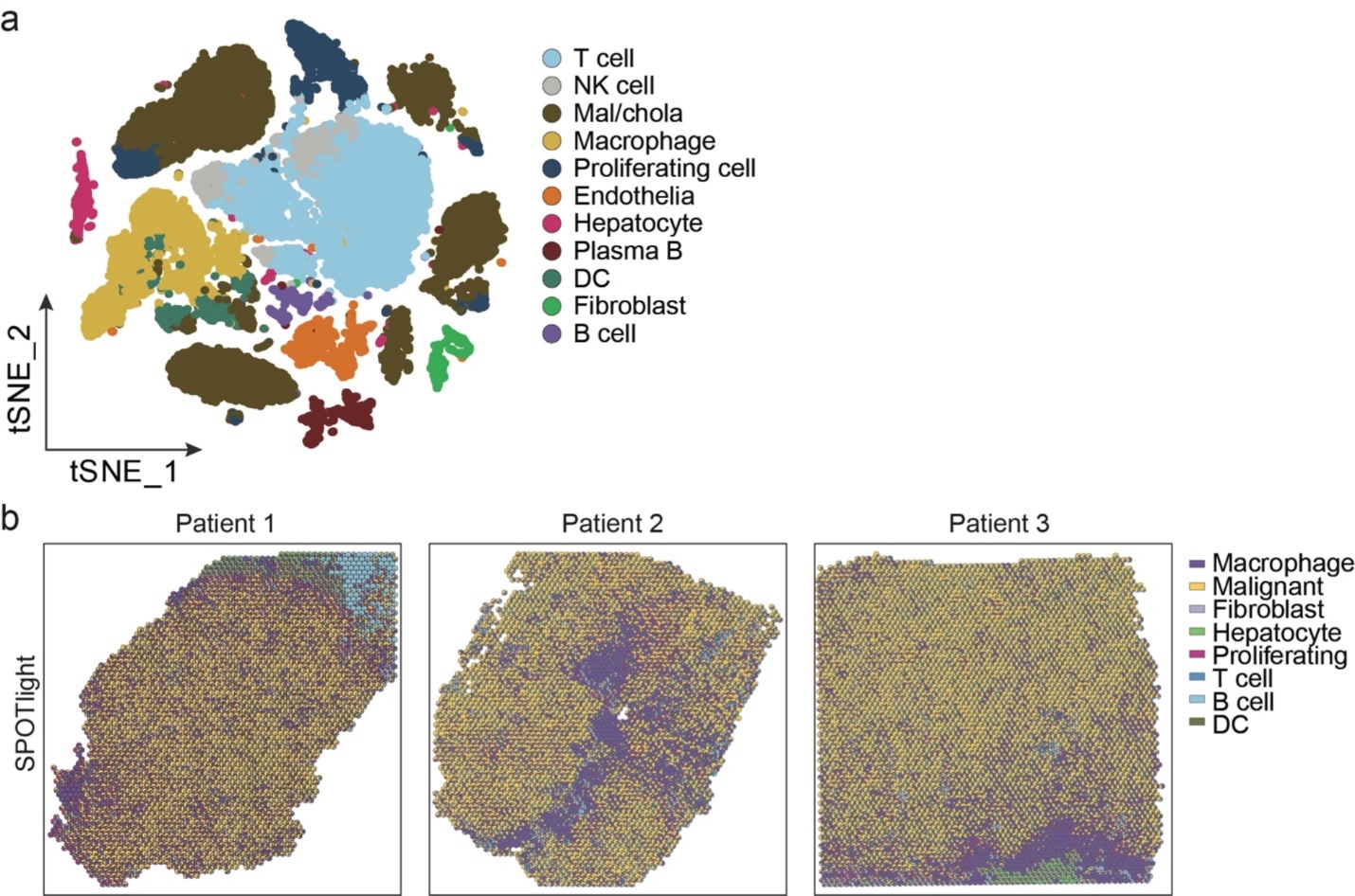


**Supplementary Fig. 2**. (a) tSNE plot showing the single cell data of GSE138709, colored by main annotated cell clusters. (b) Deconvolution of the ST spots using the single cell RNA-seq data with *SPOTlight* tool.

**
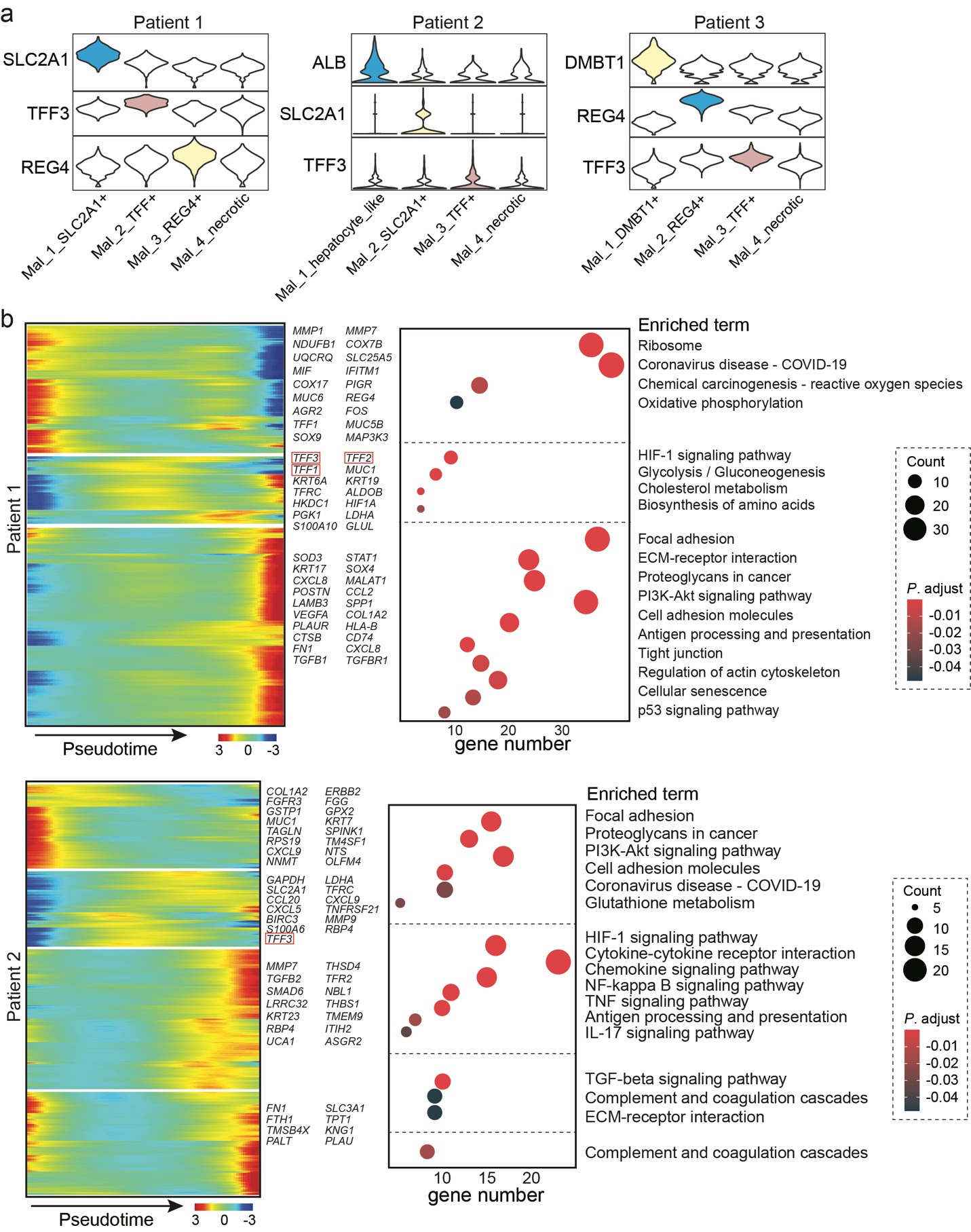
**

**Supplementary Fig. 3**. (a) Violin plots showing the expression levels of marker genes of each malignant sub-population. (b) Genes varying along the pseudo-time and the enriched signaling pathways for patient 1 and 2.

**
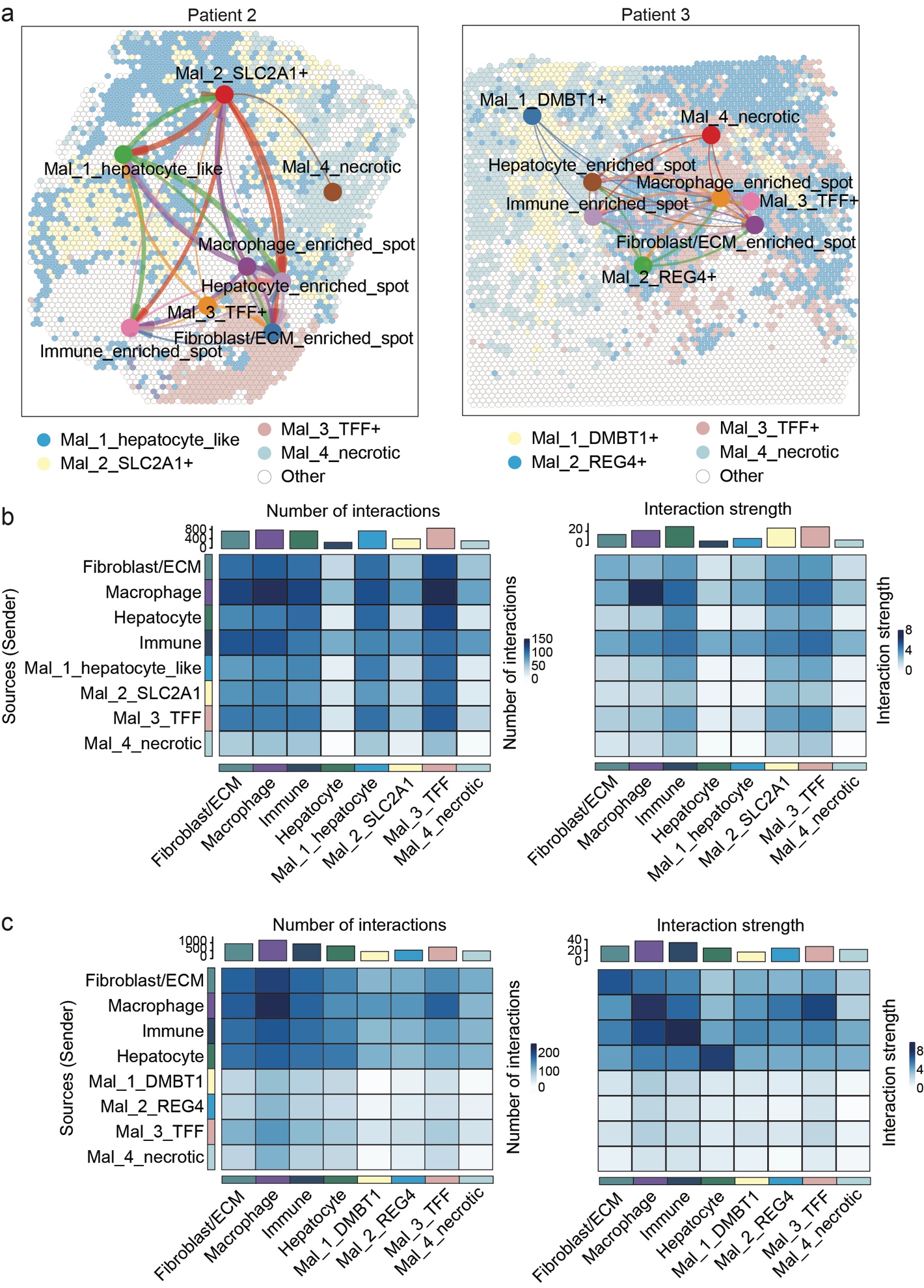
**

**Supplementary Fig. 4**. (a) Interactions between every two spot groups for patient 2 and 3. Interaction number and strength between every two spot groups for patient 2 (b) and 3 (c).


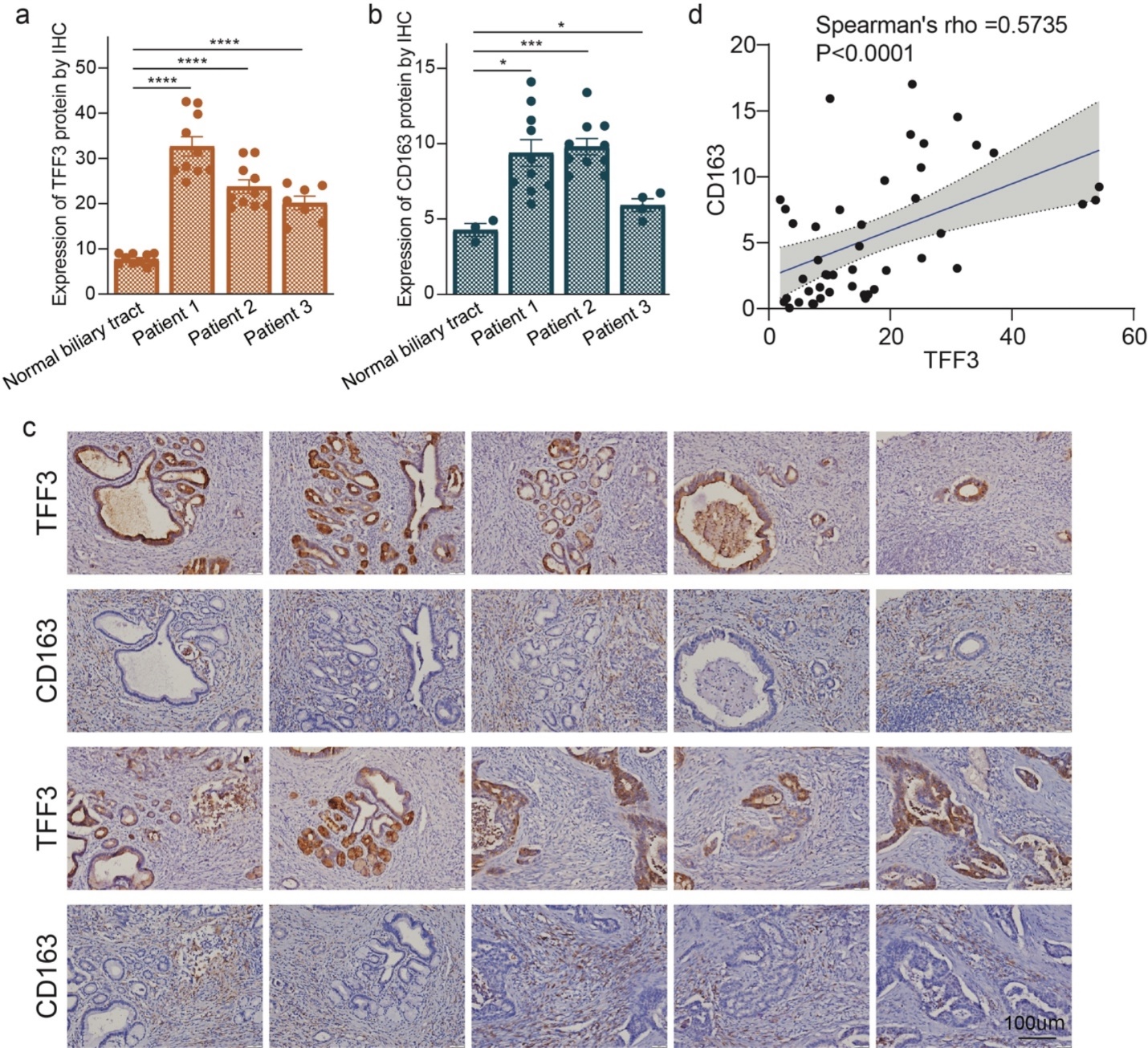


**Supplementary Fig. 5**. (a) Quantification of the expression of TFF3 represented by the number of TFF3 positive malignant cells under randomly selected microscopic views. (b) Quantification of the expression of CD168 represented by the number of CD168 positive TAMs under randomly selected microscopic views. (c) IHC staining showing the physical proximity distributions of CD163 and TFF proteins in the same or adjacent microscopic views. (d) The correlation between TFF3 and CD163 in 16 patients with TFF3 positive iCCA.


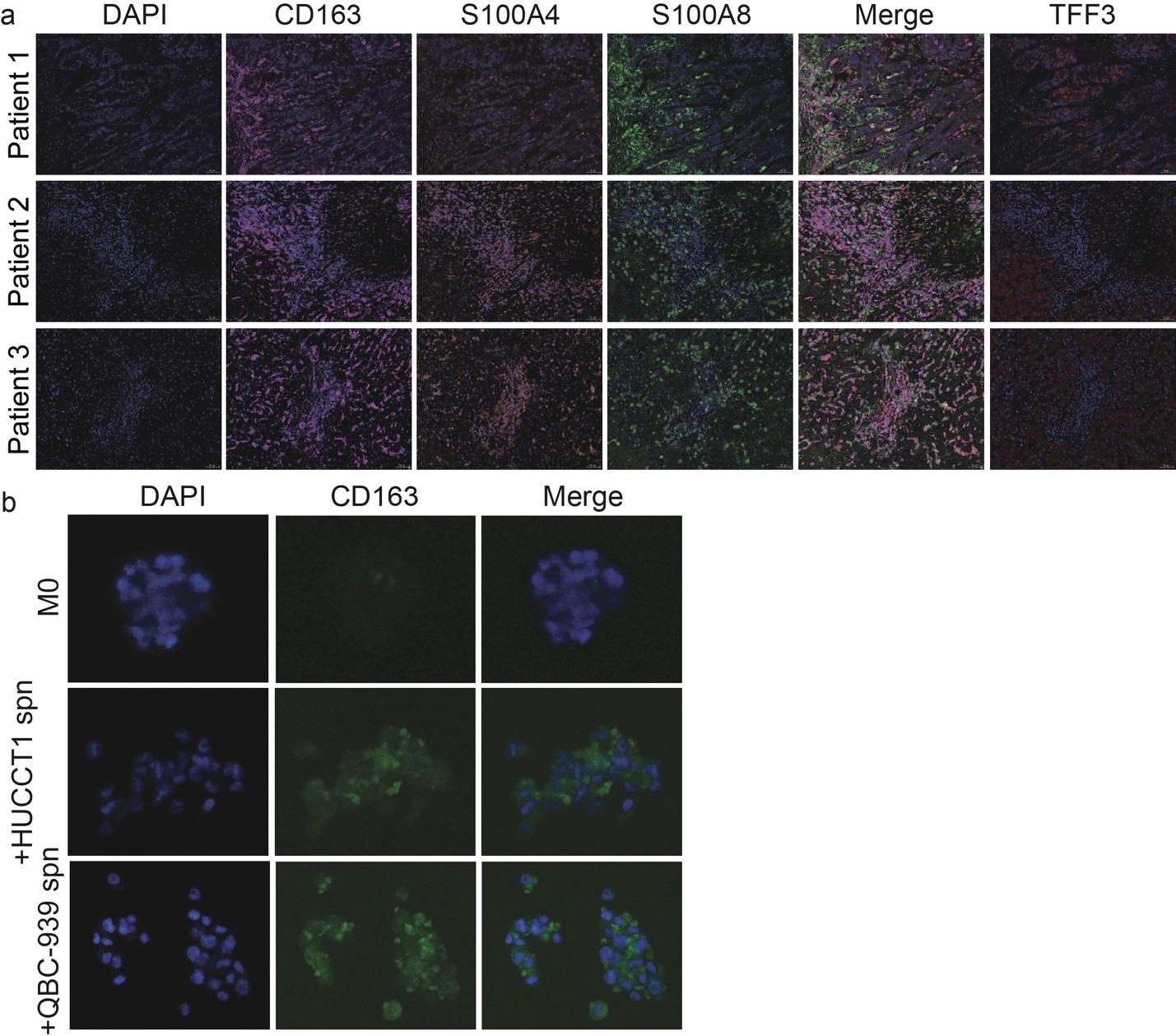


**Supplementary Fig. 6**. (a) Single channel image for multiplexed IF staining of CD163, S100A4, S100A8 and TFF3. **(b)** IF staining of CD163 after co-culture of cholangiocarcinoma cells with M0 cells.
