# Supplementary material for: Spatial resolved transcriptomics reveals distinct cross-talk between cancer cells and tumor-associated macrophages in intrahepatic cholangiocarcinoma
Source: Biomark Res. 2024 Sep 11;12:100. doi: 10.1186/s40364-024-00648-z (PMC11389341; doi:10.1186/s40364-024-00648-z)
Supplement: Supplementary file 2 — Supplementary Material 2 [file 40364_2024_648_MOESM2_ESM.docx]

| **Primer Name** | **Sequence** (5'to 3') | **Length** |
| --- | --- | --- |
| Homo-TFF3-160F | GCTCTGCTGAGGAGTACGTG | 20 |
| Homo-TFF3-160R | GGCTTGAAACACCAAGGCAC | 20 |
| Homo-ARG1-110F | TGGAAGTGAACCCATCCCTG | 20 |
| Homo-ARG1-110R | GTGATTACCCTCCCGAGCAA | 20 |
| Homo-CD206(MRC1)-124F | GATTGCAGGGGGCTTATGGG | 20 |
| Homo-CD206(MRC1)-124R | CGGACATTTGGGTTCGGGAG | 20 |
| Homo-IL10-177F | AGAACCAAGACCCAGACATCAAG | 23 |
| Homo-IL10-177R | TGGCTTTGTAGATGCCTTTCTC | 22 |
| Homo-CD163-200F | TCTCTTGGAGGAACAGACAAGG | 22 |
| Homo-CD163-200R | CCTGCACTGGAATTAGCCCA | 20 |
| Homo -S100A4-119 F | ACAGCAACAGGGACAACGAG | 20 |
| Homo -S100A4-119 R | TCATTTCTTCCTGGGCTGCTT | 21 |
| Homo-CD68-140F | CACCTCCAAGCCCAGATTCA | 20 |
| Homo-CD68-140R | GAGAGAAGCAGGTGGGGATG | 20 |
| Homo-S100A8-133F | TGCTAGAGACCGAGTGTCCT | 20 |
| Homo-S100A8-133R | GCCACGCCCATCTTTATCAC | 20 |
| Homo-TFF3_siRNA-1 | ACGUGGGCCUGUCUGCAAA/TT | 21 |
|  | UUUGCAGACAGGCCCACGU/TT | 21 |
| Homo-TFF3_siRNA-2 | GGAGUGCCUUGGUGUUUCA/TT | 21 |
|  | UGAAACACCAAGGCACUCC/TT | 21 |
| Homo-TFF3_siRNA-negative control | UUCUCCGAACGUGUCACGU/TT | 21 |
|  | ACGUGACACGUUCGGAGAA/TT | 21 |
| Homo-GAPDH_siRNA-positive control | GUAUGACAACAGCCUCAAG/TT | 21 |
|  | CUUGAGGCUGUUGUCAUAC/TT | 21 |

**Supplemental Table 1.** Primer sequence used for qPCR
